# Simplifying [^18^F]GE-179 PET: are both arterial blood sampling and 90-min acquisitions essential?

**DOI:** 10.1186/s13550-018-0396-2

**Published:** 2018-06-11

**Authors:** Colm J. McGinnity, Daniela A. Riaño Barros, William Trigg, David J. Brooks, Rainer Hinz, John S. Duncan, Matthias J. Koepp, Alexander Hammers

**Affiliations:** 1 Division of Brain Sciences, Imperial College London, London, UK; 2 MRC Clinical Sciences Centre, London, UK; 3 School of Biomedical Engineering and Imaging Sciences, King’s College London, 4th Floor Lambeth Wing, St Thomas’ Hospital, Westminster Bridge Road, London, SW1 7EH UK; 4 King’s College London and Guy’s and St Thomas’ PET Centre, St. Thomas’ Hospital, London, UK; 5 GE Healthcare Ltd, Buckinghamshire, UK; 6 Department of Nuclear Medicine, Aarhus University, Aarhus, Denmark; 7 Institute of Neuroscience, University of Newcastle, Newcastle upon Tyne, UK; 8 Wolfson Molecular Imaging Centre, University of Manchester, Manchester, UK; 9 Department of Clinical and Experimental Epilepsy, UCL Institute of Neurology, London, UK; 10 Epilepsy Society, Buckinghamshire, UK; 11 The Neurodis Foundation, CERMEP—Imagerie du Vivant, Lyon, France

**Keywords:** NMDA, PET, Compartmental modelling, CNS-5161, SUV

## Abstract

**Introduction:**

The NMDA receptor radiotracer [^18^F]GE-179 has been used with 90-min scans and arterial plasma input functions. We explored whether (1) arterial blood sampling is avoidable and (2) shorter scans are feasible.

**Methods:**

For 20 existing [^18^F]GE-179 datasets, we generated (1) standardised uptake values (SUVs) over eight intervals; (2) volume of distribution (*V*_T_) images using population-based input functions (PBIFs), scaled using one parent plasma sample; and (3) *V*_T_ images using three shortened datasets, using the original parent plasma input functions (ppIFs).

**Results:**

Correlations with the original ppIF-derived 90-min *V*_T_s increased for later interval SUVs (maximal *ρ* = 0.78; 80–90 min). They were strong for PBIF-derived *V*_T_s (*ρ* = 0.90), but between-subject coefficient of variation increased. Correlations were very strong for the 60/70/80-min original ppIF-derived *V*_T_s (*ρ* = 0.97–1.00), which suffered regionally variant negative bias.

**Conclusions:**

Where arterial blood sampling is available, reduction of scan duration to 60 min is feasible, but with negative bias. The performance of SUVs was more consistent across participants than PBIF-derived *V*_T_s.

**Electronic supplementary material:**

The online version of this article (10.1186/s13550-018-0396-2) contains supplementary material, which is available to authorized users.

## Introduction

*N*-Methyl-d-aspartate (NMDA) receptors for l-glutamate, the major excitatory neurotransmitter in the central nervous system, are ligand- and voltage-gated ion channels [1]. Receptor activation is necessary for learning while abnormal activation is associated with neurological and psychiatric disease [1].

We reported the first-in-human use of the NMDA receptor-selective PET radiotracer, [^18^F]GE-179 [2], an analogue of the non-competitive antagonist, [^11^C]CNS-5161 [3]. Quantification of [^18^F]GE-179 volume of distribution (*V*_T_) was based on 90-min scans and compartmental modelling within regions of interest (ROIs) and using rank-shaping regularisation of spectral analysis (SA) at the voxel level. [^18^F]GE-179 binding in rats and non-human primates could not be blocked in one recent study [4], but interpretation is complicated by the use of anaesthesia with the NMDA receptor inhibitors isoflurane [5] ± ketamine. We have reported substantial global changes in [^18^F]GE-179 *V*_T_ in patients with focal epilepsy, using “classical” SA [6], which is more widely used than rank-shaping.

Several ongoing studies are using [^18^F]GE-179 PET. Wide utilisation of [^18^F]GE-179 is limited by the need for arterial blood sampling and 90-min scans. There is no suitable reference region devoid of NMDA receptors. To facilitate widespread use, we report our investigations into (1) whether arterial blood sampling is avoidable, via the use of standardised uptake values (SUVs) or population-based input functions (PBIFs) and (2) whether shorter scans are feasible.

## Materials and methods

Data had been acquired previously after regulatory approvals had been obtained [2, 6]. All participants provided written, informed consent prior to participation.

Arterial blood and dynamic [^18^F]GE-179 emission scan data were available for nine healthy participants without history of neurological or psychiatric illness and 11 participants with focal epilepsy [2, 6].

Ninety-minute dynamic emission images were acquired using a Siemens/CTI ECAT EXACT HR+ (“962”) camera (Knoxville, TN, USA [7]) in 3D mode. Each participant had been injected with a smooth bolus intravenous injection of mean ± standard deviation 187 ± 4 MBq [^18^F]GE-179, at 30 s after image acquisition commenced. A rotating ^68^Ge rod source was used to acquire 10-min transmission scans for attenuation and scatter correction, prior to the dynamic emission scans. Continuous arterial blood sampling (5 ml/minute) was performed from 0 to 15 min, and a total of nine discrete arterial blood samples were taken between baseline and 90.5 min post-injection.

### MRI and PET image pre-processing

An 83-region grey-matter-only ROI map was produced for each participant using multi-atlas propagation with enhanced registration (MAPER) [8]. MAPER is an automated anatomical segmentation method that involves co-registering the participant’s T1-weighted magnetic resonance image (MRI) to that of 30 healthy controls that have already been manually labelled (the atlases). This yields individual anatomical segmentations that are fused via vote-rule decision [9] to produce the ROI map (see Additional file 1: Figure S2).

PET data pre-processing and the generation of parent plasma input functions were performed as previously described [2, 6]. Emission data had been reconstructed using Fourier rebinning (FORE [10]) and 2D-filtered backprojection (FBP; ramp filter kernel 2.0 mm full width at half maximum).

The fraction of plasma radioactivity attributable to the parent [^18^F]GE-179 had been fitted to a sigmoid function normalised to unity at 0 min using CLICKFIT in-house software version 1.7 (Hinz R, Cunningham VJ, Imaging Research Solutions Limited, London, UK) running in MATLAB 2014a (The MathWorks, Natick, MA, USA.).

The original, continuous parent plasma input functions (ppIFs) were derived as follows [11]: (1) cross-calibration of continuous and discrete whole blood radioactivity concentrations, (2) multiplication of the cross-calibrated continuous (0–15 min) whole blood radioactivity concentrations by the plasma-over-whole-blood ratio, (3) spline interpolation of the continuous (0–15 min) plasma radioactivity concentrations curve over the additional discrete measurements, and (4) multiplication of the continuous (0–90 min) plasma radioactivity curve by the parent fraction.

### Voxelwise SA

Voxelwise “classic” SA [12] was performed using Piwave 8.0 [13], using time constants of 5 s (fast boundary = 0.2 s^−1^) and 5100 s (slow boundary = 0.000196 s^−1^). We confirmed the suitability of “classic” voxelwise SA by demonstrating that the resultant original ppIF-derived *V*_T_s were strongly correlated with original ppIF-derived *V*_T_s derived from compartmental modelling (Additional file 1).

### Voxelwise SUVs

SUV images (see [11]) were calculated for all 10-min epochs from 20–30 to 80–90 min from decay-corrected summation images, using MICKPM (version 5.4 software, running in MATLAB (The MathWorks Inc., Cambridge, UK)). “MICKPM” (Modelling, Input functions and Compartmental Kinetics—Parametric Maps) is a quantitative PET analysis software that is available on request from Rainer Hinz (Wolfson Molecular Imaging Centre, University of Manchester; rainer.hinz@manchester.ac.uk). Epochs of 30–60 and 60–90 min were also tested, but yielded very similar results (data not shown).

### PBIFs

The generation of PBIFs can be split into “magnitude normalisation” and “scaling” stages, described below.

After controlling for weight and injected dose, we identified a near-significant (*p* = 0.08) negative (*r* = − 0.66) correlation between age and area under the parent fraction curve. We therefore incorporated this additional variable into the method used to generate the “magnitude normalised” input functions: (1) normalisation of participants’ ppIFs according to weight, then injected dose, then age (multiplying each participant’s ppIF according to the ratio [median value/participant value]); (2) alignment of each ppIF peak to peak at 80 s; and (3) calculation of the median (i.e. standardised) ppIF from the 19 ppIFs.

In order to generate individualised PBIFs, the median ppIF was scaled as follows: (1) linear regression of the area under the 19 other participants’ ppIF curves (AUCs) with the parent radioactivity concentration in plasma at 90 min post-injection (assumed equivalent to venous plasma [14]) and then (2) scaling of the median ppIF according to the ratio of the AUC predicted (according to the linear regression) by the parent radioactivity concentration in the participant’s arterial plasma at 90 min to the AUC of the median ppIF.

Our PBIF method depended on a single parent plasma sample. Other approaches to scaling, i.e. according to injected dose and/or body mass (kg) alone or in combination, were found to yield weaker correlations with original ppIF-derived *V*_T_s (data not shown).

The PBIFs were used to perform voxelwise SA, as above.

### Scan shortening

Voxelwise SA was performed using the original ppIFs and dynamic emission data over *t* = 0–60, t = 0–70 min, and *t* = 0–80 min, using slow component boundaries of 0.000290, 0.000256, and 0.000222 s^−1^, respectively.

### Statistical analyses

We calculated the mean (original ppIF derived or PBIF derived, as applicable) *V*_T_/SUV within seven cortical and subcortical bilateral ROIs using Analyze 7.0 (AnalyzeDirect Inc., Kansas, USA): cerebelli, hippocampi, occipital lobes, occipito-temporal (fusiform) gyri, parahippocampal gyri, putamina, superior frontal gyri (SFG), and thalami. Correlations were interrogated using Spearman’s rank coefficient. Pooled correlations were quantified using 140 data points (20 participants × seven ROIs) per variable. Original ppIF-derived and PBIF-derived *V*_T_s, and separately original ppIF-derived *V*_T_s that were calculated using 90- or 60-min datasets, were compared using Bland–Altman plots. Binding estimates were compared between subgroups [6] (epilepsy not on antidepressants, epilepsy on antidepressants, controls) by multivariate analysis of variance, with age as a covariate; a significant effect (*p* = 0.007 overall; individual ROIs *p* < 0.001) of subgroup on original ppIF-derived 90-min *V*_T_s was already reported [6].

## Results

### Voxelwise SUVs

**Fig. 1:**
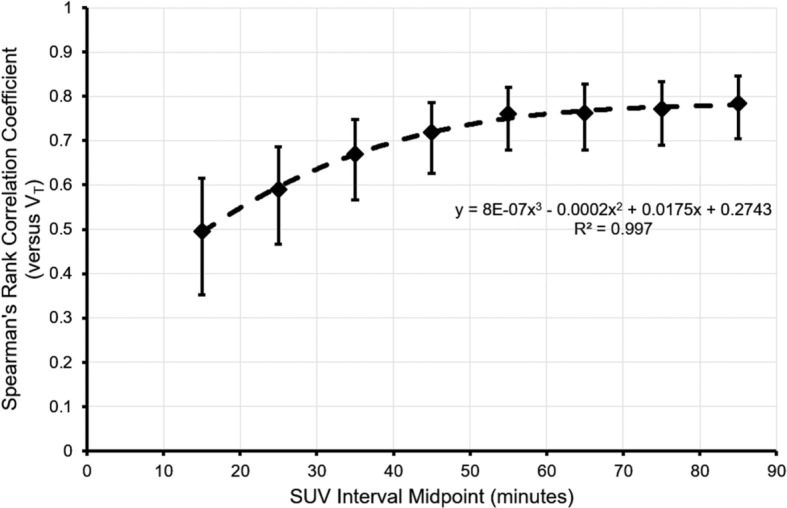
Spearman’s rank correlation coefficient versus the midpoint of the SUV interval. The correlation coefficient refers to SUV versus original ppIF-derived *V*_T_ for seven ROIs pooled. Error bars indicate 95% confidence intervals

SUVs were correlated with 90-min original ppIF-derived *V*_T_s derived from SA (seven ROIs pooled: increasing with time and reaching a maximum of *ρ* = 0.78 for 80–90 min; all *p* < 0.001; Fig. 1; Additional file 1).

The ranges of *ρ* were 0.34 (SFG) to 0.60 (cerebelli) for the interval 10–20 min, and 0.65 (hippocampi) to 0.79 (cerebelli and occipital lobes; all *p* ≤ 0.001) for the interval 80–90 min.

There was very little difference (< 1.3 percentage points) in the ROI between-subject coefficients of variation (BS-CVs) between the 80–90 min SUVs and the original ppIF-derived *V*_T_s (Additional file 1: Table S1). Larger differences were seen using earlier intervals.

The influence of subgroup on original ppIF-derived *V*_T_ [6] was not replicated for SUV data (*p* ≥ 0.05 for each interval).

### PBIFs

**Fig. 2:**
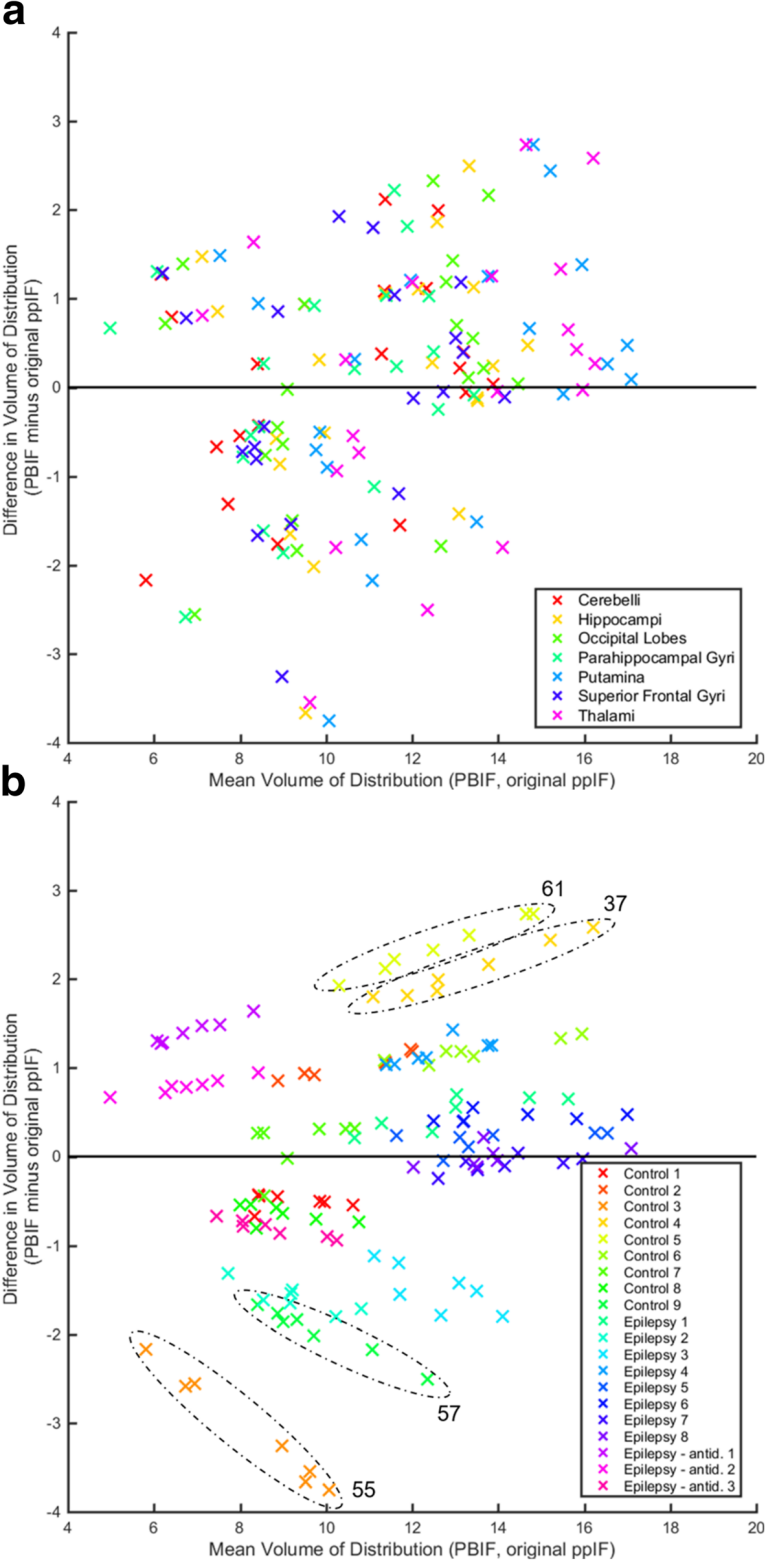
Bland–Altman plot for *V*_T_ calculated using PBIFs versus using the original ppIFs. *V*_T_s were calculated using 90-min datasets. The colour scale depicts ROI (**a**, top) or participant identification (**b**, bottom). The dash ovals identify participants with large differences in *V*_T_, with their age in years (the median age of participants was 35 years, interquartile range 26–50 years, range 20–62 years). antid., on antidepressants

PBIF-derived *V*_T_s were correlated with *V*_T_s calculated using the participants’ original ppIFs (seven ROIs pooled *ρ* = 0.90, *p* < 0.001). A small bias toward overestimation of PBIF-derived *V*_T_ was observed (linear regression *y* = 1.02*x* − 0.20; see Fig. 2) with a large variability (mean overestimate 0.7% ± 13.4%).

The range of *ρ* was 0.81 (hippocampi and occipital lobes) to 0.92 (cerebelli; all *p* < 0.001; see Additional file 1: Table S2). The absolute percent difference between PBIF-derived V_T_s and those calculated using the participants’ original ppIFs varied somewhat unpredictably across ROIs (Fig. 2), but was worse for elderly, healthy control participants.

An increase in BS-CV was observed that ranged from 2.5 percentage points (SFG) to 4.1 percentage points (occipital lobes; see Additional file 1: Table S2), i.e. mean ± standard deviation BS-CV was 26 ± 2% compared to 23 ± 2% for original ppIF-derived *V*_T_s.

The influence of subgroup on original ppIF-derived *V*_T_ [6] was not replicated for PBIF-derived *V*_T_s (*p* = 0.11).

### Scan shortening

**Fig. 3:**
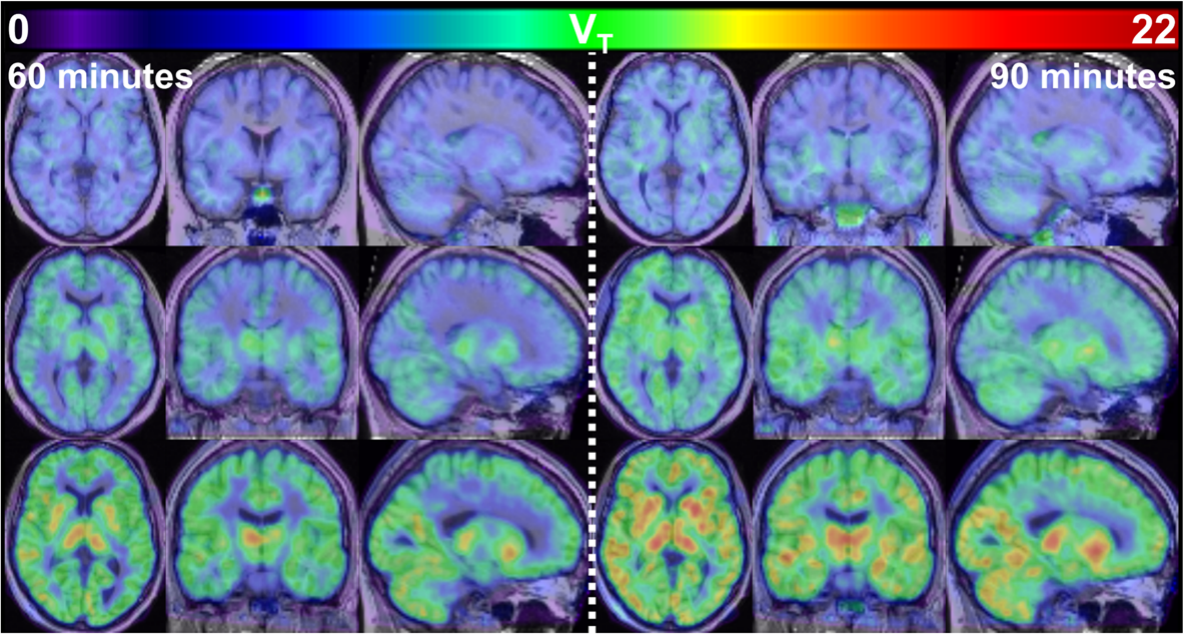
Original ppIF-derived *V*_T_ images calculated using 60-min (left panel) and 90-min (right panel) datasets. Colour scale—original ppIF-derived *V*_T_; top row—participant with epilepsy on antidepressants (epilepsy—antid. 2); middle row—control participant (control 2); bottom row—participant with epilepsy, not on antidepressants (epilepsy 5). Images are shown in radiological orientation

ROI original ppIF-derived V_T_s calculated using 60-, 70-, and 80-min datasets were positively correlated with original ppIF-derived *V*_T_s derived from the full 90-min dataset (seven ROIs pooled *ρ* = 0.98, *ρ* = 0.99, and *ρ* = 1.00 respectively; all *p* < 0.001; Table 1, Fig. 3).

**Table 1:** Original ppIF-derived *V*_T_ calculated over various intervals versus original ppIF-derived *V*_T_ calculated using the 90-min datasets, via voxelwise SA

	0–60 min			0–70 min			0–80 min			0–90 min
	Mean ± SD	*ρ*	%	Mean ± SD	*ρ*	%	Mean ± SD	*ρ*	%	Mean ± SD
Cerebelli	9.3 ± 2.4	1.00	− 6.7	9.6 ± 2.5	1.00	− 4.4	9.8 ± 2.5	1.00	− 2.2	10.0 ± 2.5
Hippocampi	9.6 ± 1.8	0.98	− 14.1	10.2 ± 2.0	0.99	− 9.0	10.8 ± 2.1	1.00	− 3.9	11.2 ± 2.2
Occipital lobes	9.9 ± 2.4	0.98	− 8.3	10.2 ± 2.5	0.98	− 5.1	10.5 ± 2.5	1.00	− 2.4	10.7 ± 2.5
Parahippocampal gyri	8.5 ± 2.0	0.98	− 13.8	9.0 ± 2.1	0.99	− 8.8	9.5 ± 2.2	0.99	− 4.1	9.9 ± 2.3
Putamina	11.7 ± 2.6	0.99	− 7.3	12.1 ± 2.7	1.00	− 4.7	12.4 ± 2.8	1.00	− 2.2	12.7 ± 2.9
Superior frontal gyri	9.4 ± 2.2	0.97	− 8.2	9.7 ± 2.3	0.98	− 5.5	10.0 ± 2.3	0.99	− 2.7	10.3 ± 2.3
Thalami	11.4 ± 2.4	0.97	− 10.1	11.9 ± 2.6	0.98	− 6.2	12.3 ± 2.6	0.99	− 2.9	12.7 ± 2.7
**Seven pooled ROIs**	**10.0 ± 2.5**	**0.98 CI** **(0.97–0.99)**	**− 9.8**	**10.4 ± 2.6**	**0.99 CI** **(0.98–0.99)**	**− 6.2**	**10.7 ± 2.6**	**1.00 CI** **(1.00–1.00)**	**− 2.9**	**11.1 ± 2.7**

*%* mean percentage difference, relative to 90-min SA original ppIF-derived *V*_T_; *ρ* Spearman’s rank correlation coefficient; *CI 95%* confidence interval; *SD* standard deviation

**Fig. 4:**
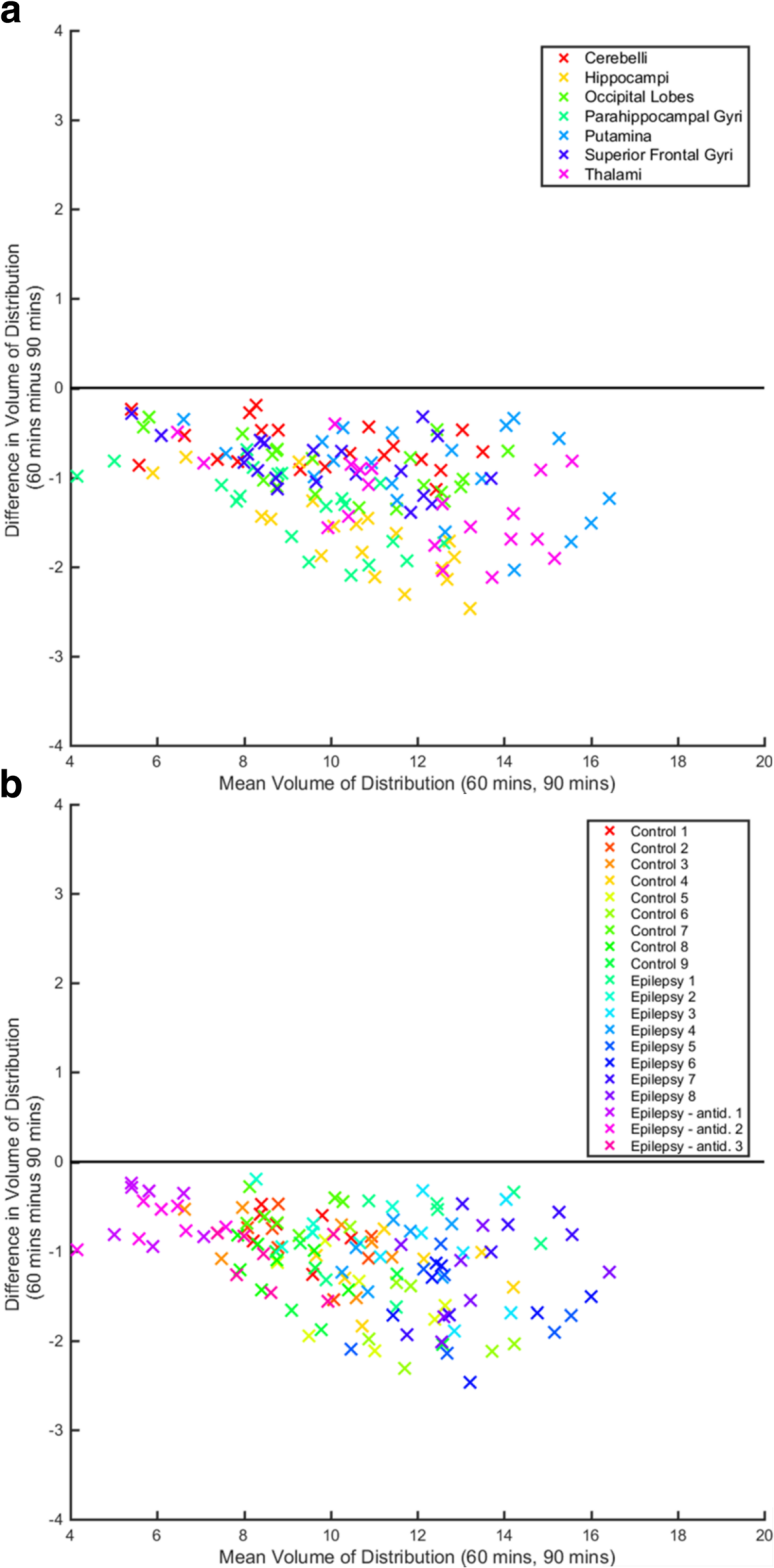
Bland–Altman plot for 60 min’ original ppIF-derived *V*_T_ versus 90 min’ original ppIF-derived *V*_T_. The colour scale depicts ROI (**a**, top) or participant identification (**b**, bottom). antid., antidepressants; mins, minutes

Correlation coefficients ranged from 0.97 (SFG and thalami, 60 min) to 1.00 (multiple ROIs and scan durations; all *p* < 0.001). The original ppIF-derived *V*_T_ was increasingly underestimated (relative to original ppIF-derived *V*_T_s calculated using 90-min datasets) with scan shortening, particularly for medial temporal lobe ROIs (Table 1, Fig. 4).

There was very little difference (< 0.9 percentage points) in ROI BS-CVs between the shortened original ppIF-derived *V*_T_s and the original ppIF-derived 90-min *V*_T_s.

The influence of subgroup on original ppIF-derived *V*_T_ [6] was replicated using all shortened datasets: 60 min (*p* = 0.001), 70 min (*p* = 0.003), and 80 min (*p* = 0.005).

## Discussion

We report further analyses of [^18^F]GE-179 PET datasets to facilitate wider use of the radiotracer. Our major findings are (1) SUVs were correlated with 90-min original ppIF-derived *V*_T_s; (2) PBIF-derived *V*_T_s were more strongly correlated with original ppIF-derived *V*_T_ estimates but had increased BS-CV; (3) original ppIF-derived *V*_T_s calculated over 60+ min were very strongly correlated with 90-min original ppIF-derived *V*_T_s, but with negative bias; and (4) we were able to replicate our original findings using the original ppIF-derived V_T_s derived from shortened datasets, but not using SUVs or PBIF-derived V_T_s.

While SUVs were only moderately correlated with original ppIF-derived *V*_T_s, the correlation coefficients started to plateau over later intervals, i.e. from 50 to 60 min onwards (see Fig. 1 and Additional file 1: Table S1). Our data suggest any increase in correlation coefficient achieved by delaying SUVs to scanning intervals beyond 90 min is likely to be modest.

PBIFs, whether scaled by injected dose, body mass, whole-blood, or plasma radioactivity, performed somewhat unpredictably across ROIs and inconsistently across participants. Although normalisation by age is not a standard approach, in practice, it had a small effect on the standardised ppIF (median difference ~ 5.5%; data not shown) which for example translated into a 0.8% difference in global PBIF-derived [^18^F]GE-179 *V*_T_. Hence, we do not believe the age normalisation procedure had a strong bearing on the results.

Where arterial blood sampling is not available, calculation of SUVs constitutes a convenient alternative with fewer outliers than the PBIF methods. However, we were unable to replicate our original finding of differences between subgroups using SUVs.

We deliberately employed a simple PBIF method that could be widely implemented. A novel method was recently described that allows simultaneous estimation of the kinetic rate constants and the input function parameters from emission data alone [15]. However, it requires a priori specification of the number of tissue compartments and can yield a similar margin of error to what we found for PBIFs [15].

It was not possible to test image-derived input functions with our data which had a limited field of view. We also could not test PBIFs anchored via venous blood samples.

Our findings suggest that where arterial blood sampling is available, it is possible to shorten the scan to 60, 70, or 80 min, without adversely affecting BS-CV or study power. However, investigators should be aware of the negative bias, which is more pronounced in the medial temporal lobe.

## Additional file

Additional file 1: Voxelwise spectral analysis (SA) versus the two-tissue compartment model (2c4kbv), voxelwise SUVs calculated overall various intervals, and PBIF-derived V_T_s. (PDF 1382 kb)
