# Supplementary material for: Simplifying [18F]GE-179 PET: are both arterial blood sampling and 90-min acquisitions essential?
Source: EJNMMI Res. 2018 Jun 11;8:46. doi: 10.1186/s13550-018-0396-2 (PMC5995767; doi:10.1186/s13550-018-0396-2)

Supplementary material for McGinnity CJ *et al.*, [^{18}F]GE-179 PET: are both arterial blood sampling and 90-minute acquisitions essential?

Voxelwise spectral analysis (SA) versus the two-tissue compartment model (2c4kbv)

In order to confirm the suitability of the more widely available voxelwise exponential spectral analysis (SA; [1]) as "ground truth" rather than the more complex rank-shaping regularisation of SA [2] which was used previously [3], we quantified the relationship between the volumes-of-distribution (V_Ts) derived from spectral analysis and V_Ts derived from the compartmental model 2c4kbv (two tissue compartments, four rate constants, variable blood volume) calculated using MICK (Modelling, Input functions and Compartmental Kinetics; version 5.2 software (available on request from Rainer Hinz, Wolfson Molecular Imaging Centre, University of Manchester, UK; rainer.hinz@manchester.ac.uk). We used the original parent plasma input functions (ppIFs) for both methods. Voxelwise SA was performed using Piwave 8.0 [4] using time constants of 5 seconds (fast component boundary=0.2 s^{-1}) and 5100 seconds slow component boundary=0.000196 s^{-1}). Here, and throughout the manuscript proper, the slow component boundary was calculated according to:

$$\beta_s = \frac{1}{t_l} \qquad (1)$$

[β_s – slow component boundary; t_l – midpoint of last frame, in seconds, post-injection].

Weighting was performed as follows [5]:

$$w_i = \frac{L_i}{T_i} \quad \textit{for frame } (i = 1, 2, 3, \ldots 34) \textit{ (non-decay corrected data)} \qquad (2)$$

[W_i – weight for frame i; L_i – length of frame i (seconds); T_i – total of true coincidences (per second) for frame i]. For ROI TACs (2c4kbv), the weights were normalised to sum(weights) = 34 (i.e. number of frames), thresholded to max(weight) ≤ 2.5, and then re-normalised to

sum(weights) = 34. For voxel TACs, weights were not normalised, but a threshold was applied according to [6, 7]:

$$\frac{\max(weight)}{\min(weight)} \leq 1000 \qquad (3)$$

Original ppIF-derived V_Ts derived from voxelwise SA were strongly correlated with original ppIF-derived V_Ts derived from regional 2c4kbv (seven ROI pooled data: $\rho = 0.93$ $p < 0.001$). Investigation of outliers (Figure S1) suggested the 2c4kbv original ppIF-derived V_T estimate was clearly erroneous for five of six outlier regions in four participants (abnormally low or high original ppIF-derived V_T, with abnormally high standard error). After exclusion of the six outliers, ρ was increased to 0.98. All SA versus 2c4kbv comparisons henceforth are after exclusion of these outliers.

Across individual ROIs, the range of ρ was 0.90 for superior frontal gyri to 0.98 for putamina (all $p < 0.001$). A small bias toward overestimation of original ppIF-derived V_T, relative to that derived from the 2c4kbv model (Figure S1), was observed (linear regression across ROIs: $y = 0.99x + 0.72$). Across ROIs, the range of median increase in original ppIF-derived V_T (SA relative to 2c4kbv) was 3.6% (hippocampi) to 8.7% (cerebelli).

Across ROIs, the range of change in between-subject coefficient of variation (BS-CV) of original ppIF-derived V_T (SA relative to 2c4kbv) was -1.8 percentage points (thalami) to -0.2 percentage points (cerebelli), i.e. spectral analysis led to slightly lower between-subject variability. The median BS-CV was 22% (range 21% – 26%) for SA, compared to 23% (20 – 26%) for 2c4kbv.

Voxelwise SUVs

TABLE S1: Voxelwise SUVs calculated overall various intervals, compared to [^{18}F]GE-179 original ppIF-derived **V_T calculated over the interval 0 – 90 minutes, via voxelwise SA.** Mean±standard deviation, between-subject coefficient of variation (BS-CV; %); Spearman's rank correlation coefficient (versus original ppIF-derived SA V_T). ppIF–parent plasma input function. SUV–standardised uptake values. V_T–volume of distribution.

	**10 – 20 Minutes**	**20 – 30 Minutes**	**30 – 40 Minutes**	**40 – 50 Minutes**	**50 – 60 Minutes**	**60 – 70 Minutes**	**70 – 80 Minutes**	**80 – 90 Minutes**	**0 – 90 Minutes ppIF-derived SA V_T**
Cerebelli	3.4 ± 0.8, 22; 0.60	3.2 ± 0.7, 23; 0.68	2.9 ± 0.7, 24; 0.75	2.7 ± 0.6, 24; 0.76	2.4 ± 0.6, 24; 0.82	2.2 ± 0.5, 25; 0.80	2.0 ± 0.5, 25; 0.79	1.8 ± 0.4, 24; 0.79	10.0 ± 2.5, 25
Hippocampi	3.0 ± 0.5, 16; 0.39	3.0 ± 0.5, 17; 0.42	2.9 ± 0.5, 17; 0.55	2.7 ± 0.4, 17; 0.54	2.5 ± 0.4, 17; 0.61	2.4 ± 0.4, 18; 0.60	2.3 ± 0.4, 19; 0.63	2.1 ± 0.4, 19; 0.65	11.2 ± 2.2, 20
Occipital lobes	3.5 ± 0.7, 20; 0.48	3.3 ± 0.7, 21; 0.64	3.1 ± 0.7, 22; 0.67	2.8 ± 0.6, 23; 0.75	2.6 ± 0.6, 24; 0.75	2.4 ± 0.6, 24; 0.77	2.2 ± 0.5, 24; 0.76	2.0 ± 0.5, 24; 0.79	10.7 ± 2.5, 23
Parahippocampal gyri	2.7 ± 0.6, 21; 0.39	2.7 ± 0.6, 21; 0.49	2.6 ± 0.6, 22; 0.54	2.4 ± 0.5, 22; 0.63	2.3 ± 0.5, 21; 0.67	2.1 ± 0.5, 22; 0.68	2.0 ± 0.4, 22; 0.70	1.9 ± 0.4, 23; 0.73	9.9 ± 2.3, 23
Putamina	4.0 ± 0.8, 19; 0.46	3.9 ± 0.8, 20; 0.56	3.6 ± 0.7, 20; 0.64	3.3 ± 0.7, 21; 0.64	3.0 ± 0.6, 21; 0.73	2.8 ± 0.6, 22; 0.71	2.6 ± 0.6, 22; 0.72	2.4 ± 0.5, 22; 0.74	12.7 ± 2.9, 23
Superior frontal gyri	3.3 ± 0.5, 16; 0.34	3.2 ± 0.5, 17; 0.48	2.9 ± 0.6, 19; 0.55	2.7 ± 0.5, 20; 0.54	2.4 ± 0.5, 21; 0.62	2.2 ± 0.5, 21; 0.65	2.0 ± 0.4, 22; 0.64	1.9 ± 0.4, 22; 0.68	10.3 ± 2.3, 23
Thalami	3.7 ± 0.7, 18; 0.48	3.6 ± 0.7, 18; 0.61	3.4 ± 0.7, 19; 0.63	3.2 ± 0.6, 20; 0.69	3.0 ± 0.6, 21; 0.72	2.8 ± 0.6, 22; 0.70	2.5 ± 0.6, 22; 0.71	2.4 ± 0.5, 22; 0.70	12.7 ± 2.7, 21
**Seven pooled ROIs:**	***3.4 ± 0.7, 22; 0.50***	***3.3 ± 0.7, 22; 0.59***	***3.1 ± 0.7, 23; 0.67***	***2.8 ± 0.7, 23; 0.72***	***2.6 ± 0.6, 23; 0.76***	***2.4 ± 0.6, 24; 0.76***	***2.2 ± 0.5, 24; 0.77***	***2.1 ± 0.5, 24; 0.78***	***11.1 ± 2.7, 24***

PBIFs

TABLE S2: PBIF-derived [^{18}F]GE-179 V_Ts compared to original ppIF-derived [^{18}F]GE-179 V_Ts. V_Ts were calculated by voxelwise spectral analysis over the interval 0 – 90 minutes. Mean±standard deviation, between-subject coefficient of variation (BS-CV; %); Spearman's rank correlation coefficient (versus original ppIF-derived SA V_T). ppIF–parent plasma input function. SUV–standardised uptake values. V_T–volume of distribution.

	**PBIF-derived 0 – 90 Minutes SA V_T**	**Original ppIF-derived 0 – 90 Minutes SA V_T**	**Percent difference (PBIF-derived minus ppIF-derived)**
Cerebelli	10.1 ± 2.9, 29; 0.92	10.0 ± 2.5, 25	0.9 ± 13.8, 1602
Hippocampi	11.2 ± 2.6, 23; 0.81	11.2 ± 2.2, 20	0.5 ± 13.7, 2704
Occipital lobes	10.9 ± 3.0, 28; 0.81	10.7 ± 2.5, 23	1.0 ± 13.8, 1415
Parahippocampal gyri	9.9 ± 2.6, 27; 0.87	9.9 ± 2.3, 23	0.7 ± 14.0, 2033
Putamina	12.8 ± 3.4, 26; 0.89	12.7 ± 2.9, 23	0.9 ± 13.4, 1509
Superior frontal gyri	10.2 ± 2.6, 25; 0.83	10.3 ± 2.3, 23	0.2 ± 13.7, 7593
Thalami	12.7 ± 3.2, 25; 0.84	12.7 ± 2.7, 21	0.7 ± 13.6, 1982
**Seven pooled ROIs:**	***11.1 ± 3.1, 28; 0.90***	***11.1 ± 2.7, 24***	***0.7 ± 13.4, 1966***

References

1. Cunningham VJ, Jones T. Spectral analysis of dynamic PET studies. J Cereb Blood Flow Metab. 1993;13(1):15-23.
2. Turkheimer FE, Hinz R, Gunn RN, Aston JA, Gunn SR, Cunningham VJ. Rank-shaping regularization of exponential spectral analysis for application to functional parametric mapping. Phys Med Biol. 2003;48(23):3819-41.
3. McGinnity CJ, Hammers A, Riano Barros DA, Luthra SK, Jones PA, Trigg W, et al. Initial evaluation of ^{18}F-GE-179, a putative PET Tracer for activated N-methyl D-aspartate receptors. J Nucl Med. 2014;55(3):423-30.
4. Turkheimer FE, Brett M, Visvikis D, Cunningham VJ. Multiresolution analysis of emission tomography images in the wavelet domain. J Cereb Blood Flow Metab. 1999;19(11):1189-208.
5. McGinnity CJ, Riano Barros DA, Rosso L, Veronese M, Rizzo G, Bertoldo A, et al. Test-retest reproducibility of quantitative binding measures of [^{11}C]Ro15-4513, a PET ligand for GABAA receptors containing alpha5 subunits. NeuroImage. 2017;152:270-82.
6. Aston J, Worsley K, Gunn R. RPM statistics — a statistical tool for receptor parametric mapping. NeuroImage. 2001;13:65.
7. Gunn RN, Sargent PA, Bench CJ, Rabiner EA, Osman S, Pike VW, et al. Tracer kinetic modeling of the 5-HT1A receptor ligand [carbonyl-^{11}C]WAY-100635 for PET. NeuroImage. 1998;8(4):426-40.
8. Ashburner J, Friston KJ. Unified segmentation. NeuroImage. 2005;26(3):839-51.

Figure titles and legends

Figure S1: [^{18}F]GE-179 original ppIF-derived **V_T calculated using voxelwise SA versus using the regional 2c4kbv model.** Bland – Altman plot. The six outliers (see text) are highlighted in dashed circles. The colour scale depicts ROI (A, top) or participant identification (B, bottom). antid.-on antidepressants.

Figure S2: MAPER-derived ROIs for a representative participant (Epilepsy 2). The participant's T1-weighted MR image is shown in the left panels, and the corresponding original ppIF-derived [^{18}F]GE-179 V_T image is shown in the right panels. The cerebelli (Cere) are delineated in yellow, the hippocampi (HC) in red, the occipital lobes (OL) in blue, the occipito-temporal (fusiform) gyri (OL) in blue, the parahippocampal gyri (PHG) in green, the putamina (Puta) in cyan, the superior frontal gyri (SFG) in orange, and the thalami (Thal) in magenta. Other regions that were segmented via MAPER in the present study are delineated in white. The ROIs were masked using the grey matter map (thresholded at 50% probability) produced by tissue class segmentation of the T1-weighted image in SPM12[8] (Statistical Parametric Mapping, Wellcome Trust Centre for Neuroimaging, University College London, London, www.fil.ion.ucl.ac.uk/spm). Images are shown in radiological orientation. MAPER – multi-atlas propagation with enhanced registration. MR-magnetic resonance. ROIs-regions of interest. V_T-volume of distribution.

Figure S1

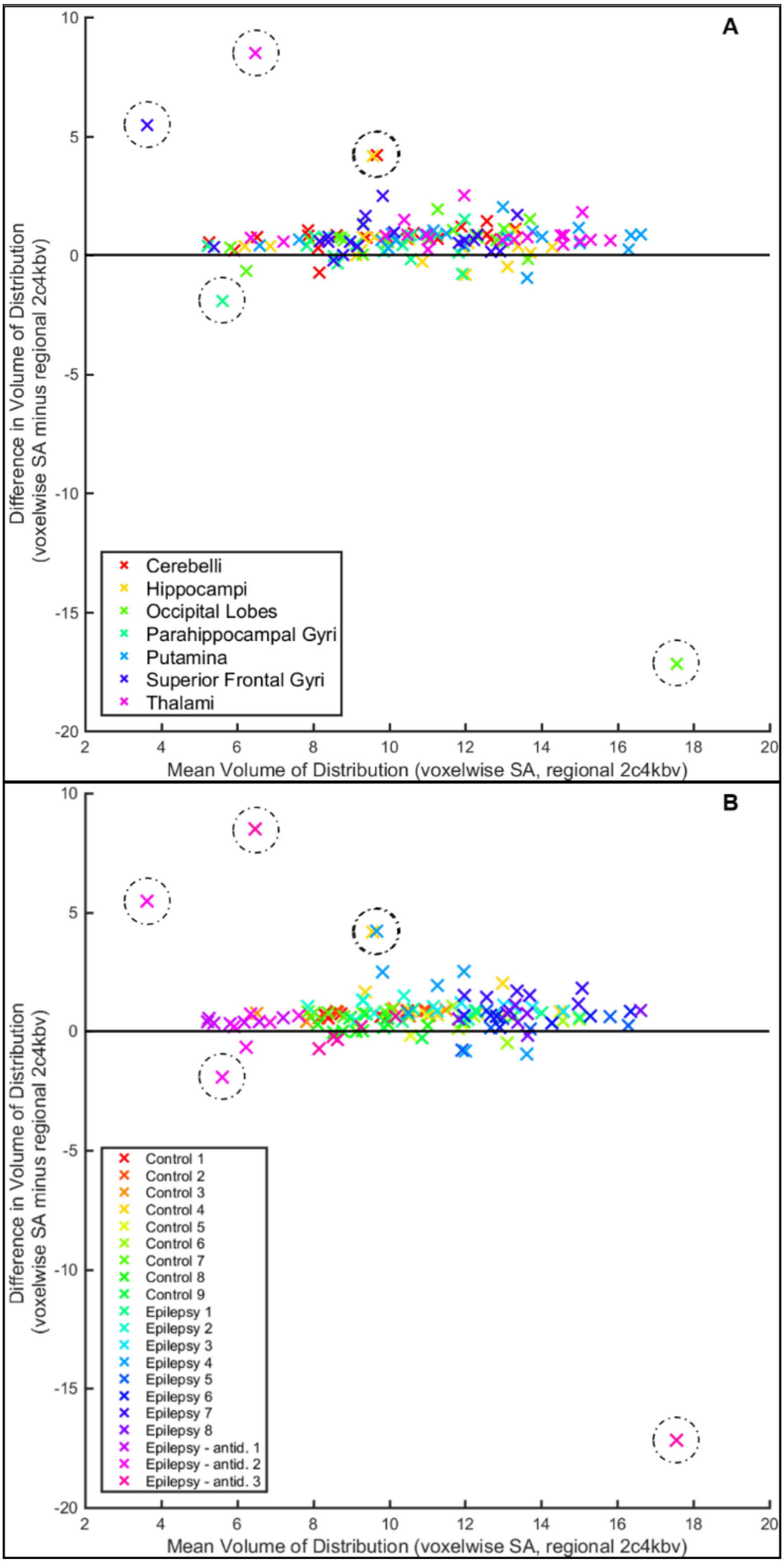
A
Cerebelli
Hippocampi
Occipital Lobes
Parahippocampal Gyri
Putamina
Superior Frontal Gyri
Thalami
Difference in Volume of Distribution (voxelwise SA minus regional 2c4kbv)
Mean Volume of Distribution (voxelwise SA, regional 2c4kbv)
B
Control 1
Control 2
Control 3
Control 4
Control 5
Control 6
Control 7
Control 8
Control 9
Epilepsy 1
Epilepsy 2
Epilepsy 3
Epilepsy 4
Epilepsy 5
Epilepsy 6
Epilepsy 7
Epilepsy 8
Epilepsy - antid. 1
Epilepsy - antid. 2
Epilepsy - antid. 3
Difference in Volume of Distribution (voxelwise SA minus regional 2c4kbv)
Mean Volume of Distribution (voxelwise SA, regional 2c4kbv)

Figure S2

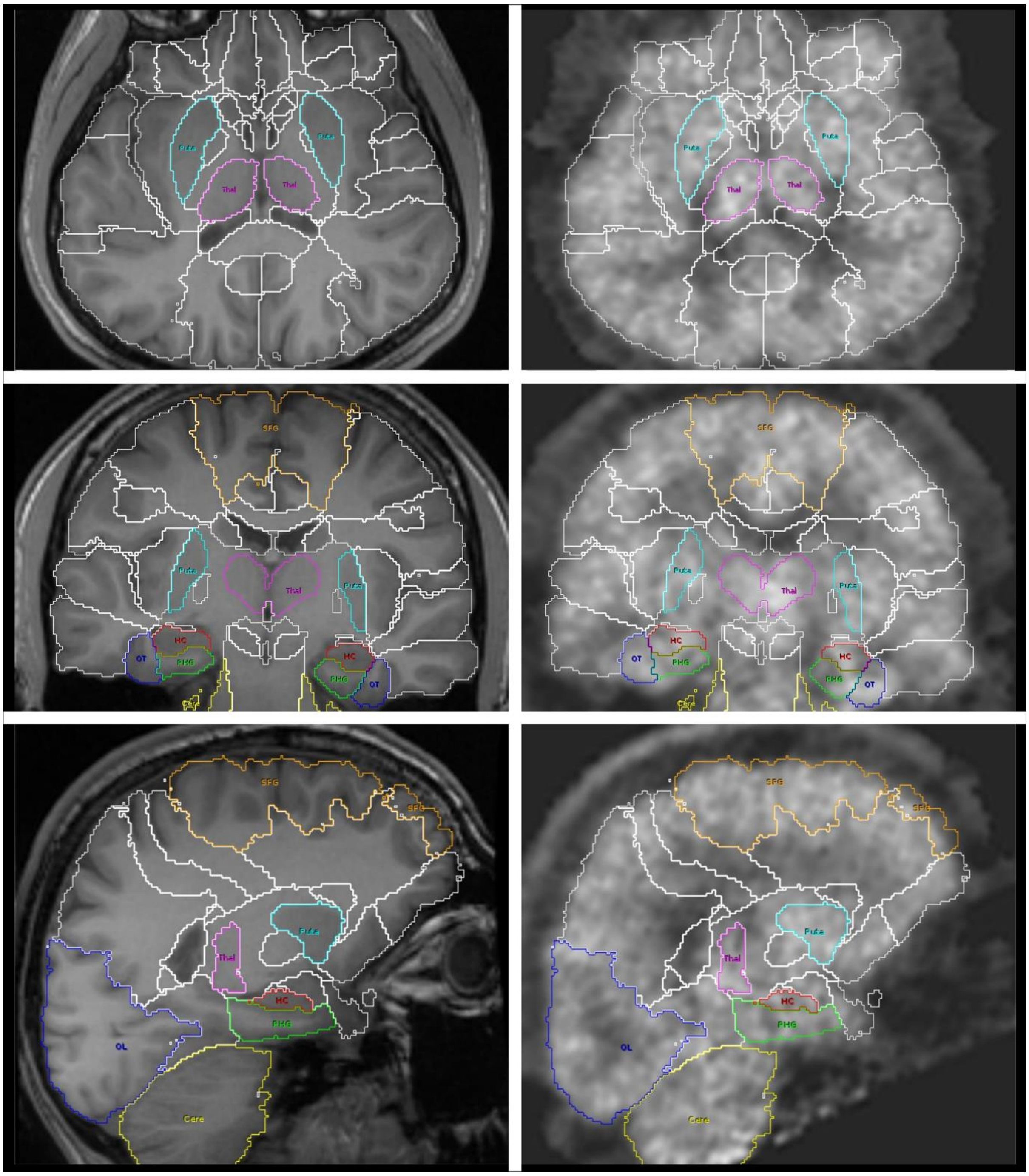

Supplement: Supplementary file 1 — Voxelwise spectral analysis (SA) versus the two-tissue compartment model (2c4kbv), voxelwise SUVs calculated overall various intervals, and PBIF-derived VTs. (PDF 1382 kb) [file 13550_2018_396_MOESM1_ESM.pdf]
